# Ultra-light antennas via charge programmed deposition additive manufacturing

**DOI:** 10.1038/s41467-024-53513-w

**Published:** 2025-01-08

**Authors:** Zhen Wang, Ryan Hensleigh, Zhenpeng Xu, Junbo Wang, James JuYoung Park, Anastasios Papathanasopoulos, Yahya Rahmat-Samii, Xiaoyu (Rayne) Zheng

**Affiliations:** 1 Advanced Manufacturing and Metamaterials Laboratory, Department of Material Science and Engineering, University of California, Berkeley, CA USA; 2 Department of Civil and Environmental Engineering, University of California, Los Angeles, CA USA; 3 Department of Electrical and Computer Engineering, University of California, Los Angeles, CA USA; 4 Lawrence Berkeley National Laboratory, Berkeley, CA USA

**Keywords:** Electronic devices, Electrical and electronic engineering

## Abstract

The demand for lightweight antennas in 5 G/6 G communication, wearables, and aerospace applications is rapidly growing. However, standard manufacturing techniques are limited in structural complexity and easy integration of multiple material classes. Here we introduce charge programmed multi-material additive manufacturing platform, offering unparalleled flexibility in antenna design and the capability for rapid printing of intricate antenna structures that are unprecedented or necessitate a series of fabrication routes. Demonstrating its potential, we present a transmitarray antenna composed of an interconnected, multi-layered array of dielectric/conductive S-ring unit cells, reducing 94% mass of conventional antenna configurations. A fully printed circular polarized transmitarray system fed by a source and a Risley prism antenna system operating at 19 GHz both show close alignment between testing results and numerical simulations. This printing method establishes a universal platform, propelling discovery of new antenna designs and enabling data-driven design and optimizations where rapid production of antenna designs is crucial.

## Abstract (web summary)

Charge programmed additive manufacturing seamlessly integrates multiple material classes and provides a universal platform for rapid printing of ultralight antennas. It propels discovery of antenna designs with unprecedented structural complexity.

## Introduction

Antennas are essential components of all radio equipment that transmit and receive energy as electromagnetic waves. Next-generation wireless communications such as 5 G/6 G^1^, Internet of Things (IoT)^2^, small satellite communications^3^, etc., necessitate lightweight, low-profile, and high-performance antennas. These emerging applications drive innovations in not only antenna design but also evolutions in antenna manufacturing techniques.

The manufacturing of antennas, depending on the designs and layouts, has been accomplished through multiple techniques, such as lithography and machining. These methods are limited in attainable geometrical complexities and require excessive structural materials such as substrate to support features, hence incurring undesirable weight. Recently, antenna designs have begun to utilize additive manufacturing (AM) to integrate 3D lattices^4–7^, corrugations, septums^8^, and corporate waveguides^9^ to improve antenna size, weight, and electromagnetic performance. However, these AM processes have also limited the designs to single materials (all-dielectric or all-metal), leaving a range of electromagnetic devices not easily achievable (e.g., transmitarrays and metasurfaces based on multi-layer conductive elements). Multi-process AM of antennas with both dielectric and metal is possible, but the complex, bespoke combination of techniques, including the requisite alignment and optimization necessary, limits its broad applicability to other or complex designs^10,11^ (see Table S1 in the supplementary material for a summary of state-of-the-art techniques for advanced antenna fabrication). Additionally, all current multi-material AM processes rely on a toolpath or a substrate to write conductive ink on^12–14^. These processes are limited in toolpath complexity and require incorporating excessive structural material weight due to limitations in printer resolution (>100 μm) and the inability of printer feedstocks to support their own weight^9,15^. To enable conductive features, these techniques have to involve high-temperature post-sintering to turn nanoparticle inks into conductive metal patterns, which are typically less conductive than bulk metals^16^ and tend to induce significant shrinkage and cracks. These limitations make antenna fabrication challenging for complex 3D electronic architectures and rule out the compatibility with dielectric materials sensitive to temperature.

Additive manufacturing of structural materials in the past decade has realized sophisticated 3D architected lattice materials with an interconnected network of truss-like unit cells, which allows designers to access materials with ultra-lightweight yet mechanically functional materials^17–20^. Translating these design concepts to produce electronics is compelling. Incorporating such concepts can significantly benefit multilayer antennas that incorporate multiple mixed layers of dielectric laminate and metallic traces, such as microstrip antenna arrays and transmitarrays. However, this requires the complex interpenetration of metal and dielectric, which is beyond what traditional lithography, metal machining, and conventional AM techniques can achieve.

**Fig. 1:**
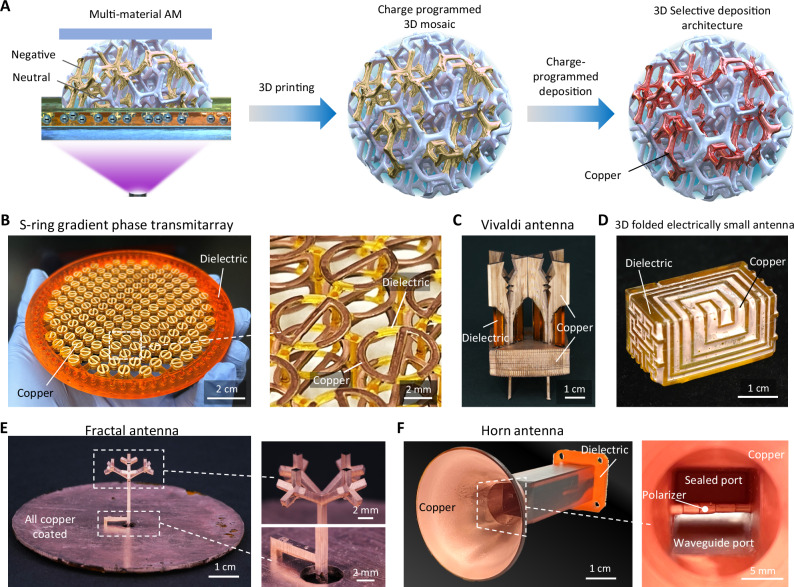
Charge programmed deposition additive manufacturing as a versatile platform for fast production of 3D antenna systems. **A** Charge programmed printing and deposition scheme. **B**–**F** Photos of charge programmed deposition additive manufactured antennas: **B** a gradient phase transmitarray with three layers of interpenetrating S-rings and dielectric materials; **C** a Vivaldi antenna; **D** a 3D folded electrically small antenna; **E** a tree fractal antenna; **F** a horn antenna with a septum polarizer.

In this work, we report charge programmed multi-material 3D printing^21^ as a versatile and universal platform for rapid production of nearly all types of 3D antenna systems. The technique allows free design space and straightforward transformation into arbitrary complex 3D layouts of metals, dielectric materials, and their interpenetrating composites with high precision. In contrast to other techniques that are each suitable for a particular antenna configuration, our technique is compatible with a wide range of antenna designs, see Fig. 1. Via patterning highly conductive metals with a wide range of dielectric materials into a 3D layout, we demonstrate the creation of a lightweight transmitarray antenna enabled by 3D architected designs comprised of multi-layer sub-wavelength conductive and dielectric elements which were previously not possible using any techniques. We then present a scalable fabrication method that significantly expands the build area to achieve industrially competitive antenna sizes with minimal loss in performance. Additionally, we demonstrate the direct printing of a lightweight septum horn antenna with complex internal channels, which is then integrated with the transmitarray to form an all-in-one antenna system. This ultralight architected antenna concept is also extended to construct a gradient-phase transmitarray panel, which enables a low-profile 2D beam steering antenna in the Risley prism configuration^22^. The fabricated transmitarray and horn achieve competitive performance with an order of magnitude less weight. These results indicate the broad and comprehensive applicability of charge-programmed 3D printing in the fabrication of advanced antennas that paves the way for the next-generation communications and allows ultra-lightweight antenna designs that were not previously possible.

## Results

### Charge programmed deposition manufacturing as a universal platform for 3D antenna printing

The charge programmed deposition (CPD) manufacturing program is based on patterning and controlling surface charge polarity via multi-material printing of photo monomers with varying pendant reactive groups. The charge-programmed 3D mosaic, combining positive, negative, and neutral charged areas, forms a patterned substrate upon which selective micro-fabrication of metallic and other functional materials can be carried out – when the sub-domain within the 3D substrate and deposition material have opposite charge polarity, there is attraction and deposition; and like polarity or no polarity (neutral) repels or gives no plating (Fig. 1A). Surface charge is achieved by blending inherently charged photo monomers into the printing ink. Here we utilize projection stereolithography (SLA) to pattern dielectric phases with prescribed polarities, due to its fine feature size (<20 μm) and large build areas (~10 cm XY footprint)^23^. The entire fabrication process has minimal steps without reliance on toolpath, post-sintering, or a substrate to write on (see Supplementary Materials Section 1 and Figs. S1 and S2).

**Fig. 2:**
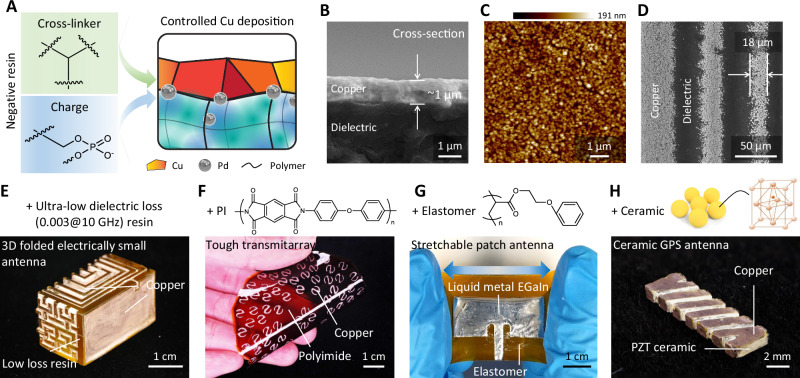
Optimization of selectivity and extension to other materials. **A** Schematic of the composition regulated copper deposition. **B** Scanning electron microscopic (SEM) image showing the cross section of copper cladding on the dielectric material. **C** Atomic force microscopic image showing the dense and smooth copper deposited on the negative resin. **D** SEM image showing the smallest feature size of CPD. **E**–**H** Demonstration of the enabled complex 3D antenna structures and compatibility with a wide range of materials: **E** a 3D folded electrically small antenna^24^ with interpenetrating metal and dielectric materials based on a commercial ultra-low dielectric loss resin, **F** polyimide (PI) with selectively patterned copper, **G** a stretchable patch antenna with liquid metal eutectic gallium-indium alloy as the conducting phase, and **H** a lead zirconate titanate (PZT) ceramic antenna for global positioning system (GPS) application^35^.

Figure 1B demonstrates the versatility of our platform for virtually all types of antennas with reduced weight. Figure 1B shows a S-ring gradient phase transmitarray fabricated by CPD for generating highly directive radiation. The antenna is featured with 3 layers of gradually tilted architected S-ring unit cells. A CPD Vivaldi antenna is shown in Fig. 1C with the dielectric and metal interpenetrating in the 3D layout. Figure 1D showcases a 3D folded implantable electrically small antenna featured with 3D interpenetrating Archimedean spirals and Hilbert curves^24^. Conventionally, transmitarray, Vivaldi, and the folded antennas were typically assembled by stacking, assembling, or folding copper clad laminates with metal patterns generated by lithography. The excessive dielectric laminate materials limit the 3D design freedoms and result in heavy weight. Figure 1E shows a printed 3D fractal antenna which would otherwise be fabricated by selective laser sintering, which typically suffers from very rough surface of the sintered bulky copper^25^. Figure 1F shows a horn antenna that integrates a septum polarizer for generating wide-band circular polarization. Patch antenna and helical antenna enabled by this technique are shown in Fig. 2. With high printing precision, simple process protocol, and vast design freedom, CPD offers a universal platform for fabricating all types of light-weight antennas which would otherwise be impossible or have to rely on multiple manufacturing routes, as summarized in Table 1.

**Table 1:** CPD fabricated antennas and their typical conventional fabrication methods

Antenna type	Lithography	Metal machining	Selective laser melting	Stereolithography/digital light processing	CPD (this work)
Transmitarray (Figs. 1B and 3)	X				X
Vivaldi antenna (Fig. 1C)	X				X
3D folded electrically small antenna (Fig. 1D)	X				X
Tree fractal antenna (Fig. 1E)	X		X		X
Horn antenna (Fig. 1F)		X	X	X	X
Patch antenna (Fig. 2G)	X				X
Helical antenna (Fig. 2H)		X			X

### Expanding tailorable materials pallets for antenna printing

High-performance 3D antennas require high-quality metal deposition to maximize the signal/noise ratio. By tuning the composition of the charged ink, we optimized the crosslink densities and charge densities of the UV sensitive resin which facilitate the selective autocatalytic process of copper patterning and ensure final metal density, conductivity and spatial resolution of the 3D patterning^26^ (see details in Materials and Methods). In order to further improve the catalytic activity of our previous work^21^, the bound Pd ions were in situ reduced into Pd metal nanoparticles embedded on the local surface of the charged area. By tuning the ratio of the crosslinker and charge monomer, we achieved controlled distribution of Pd in both the particle size and the embedded depth into the charged surface (Fig. 2A). This consequently ensured efficient catalysis of the following copper deposition being free-of-cracks, dense, smooth, and uniform as shown in Fig. 2B, C. The deposited copper had a very high conductivity of 4.9 × 10^7^ S m^−1^ which is comparable to that of annealed copper, 5.8 × 10^7^ S m^−1^, promising its performance in antenna application. This technique achieved a fine feature size of 18 µm for the patterned metal (Fig. 2D), which is identical to the pixel size of the digital micro-mirror device chip in the projection stereolithography system.

CPD can broadly integrate with a variety of multi-material 3D printing methods^27^. CPD allows essentially any complex 3D structure, including complex lattices, and has demonstrated deposition of copper with near-pristine conductivity, magnetic materials (Fe_3_O_4_), semiconductors (ZnO), nanomaterials (CNTs, etc.), and combinations of these via multiple depositions onto the underlying 3D photopolymer substrate. Additionally, by blending high-κ powders with the photopolymer, we can vary the dielectric constant continuously from the ultra-low (~2) of neat acrylates to ultra-high (>800)^21^. Particularly for antennas, this approach opens up exciting opportunities to realize antenna topology designs that cannot be realized by any current 3D printing/fabrication methods, such as Vivaldi antenna arrays^28^ featured with broadband characteristics and other antennas with different features (Fig. S2).

For the dielectric phase, we extended charge programmable materials from stiff polymers to a variety of other materials that are attractive for many other antenna application scenarios. Via introducing the negative charge monomer bis(2-(methacrylooyloxy)ethyl)) phosphate (PDD) into a commercial ultra-low dielectric loss resin, polyimide precursors, epoxy resins, flexible acrylates, and ceramic resins, we show in Figs. 2E to 2H and S3 materials compatible with our printing technique, including high dielectric performance and tough polymers, elastomers, and high-temperature ceramics (see Materials and Methods for details). The negative resins were printed with each of their corresponding neutral counterparts to guide the selective metal deposition. Figure 2E shows an example of a folded electrically small antenna with 3D metal patterns printed using a commercial ultra-low dielectric loss resin which is favored for high-frequency applications. Neutral polyimide (also known as “Kapton”) has been successfully 3D printed by complexing its precursor polyamic acid with a photo curable acrylate with an amine group^29^. We blended PDD into the neutral resin to get the negative resin. Using CPD, we achieved selective copper deposition fabricating a transmitarray on polyimide (Fig. 2F) which has dielectric properties and mechanical properties (Young’s modulus 2.4 ± 0.2 GPa, Fig. S3A) that can serve in extreme temperatures ranging from − 269 °C to + 400 °C, X-ray conditions, and space environment. The charge monomer PDD can also introduce polarity into a 3D printable thermoset epoxy resin^30^ and we fabricated a patch antenna with good chemical and structural stability (Fig. S3B). Blending PDD with a mono functional acrylate which self-coils the polymer chains due to the large stereo hindrance, we formulated resins for CPD of copper on an elastomer (Fig. 2G). To match the large elastic deformation, liquid metal gallium-indium eutectic (EGaIn) that can selectively wet the copper surface was applied to demonstrate stretchable and wearable devices^31,32^. Not only with polymer systems, our method is also compatible with ceramics. Introducing PDD into a printable neutral ceramic resin^33,34^, we got a negative ceramic resin and printed a ceramic global positioning system (GPS) antenna^35^, as shown in Fig. 2H, which has excellent dielectric stability and can withstand ultrahigh temperatures ( ~ 1000 °C).

### Design and scalable fabrication of ultralight transmitarray

**Fig. 3:**
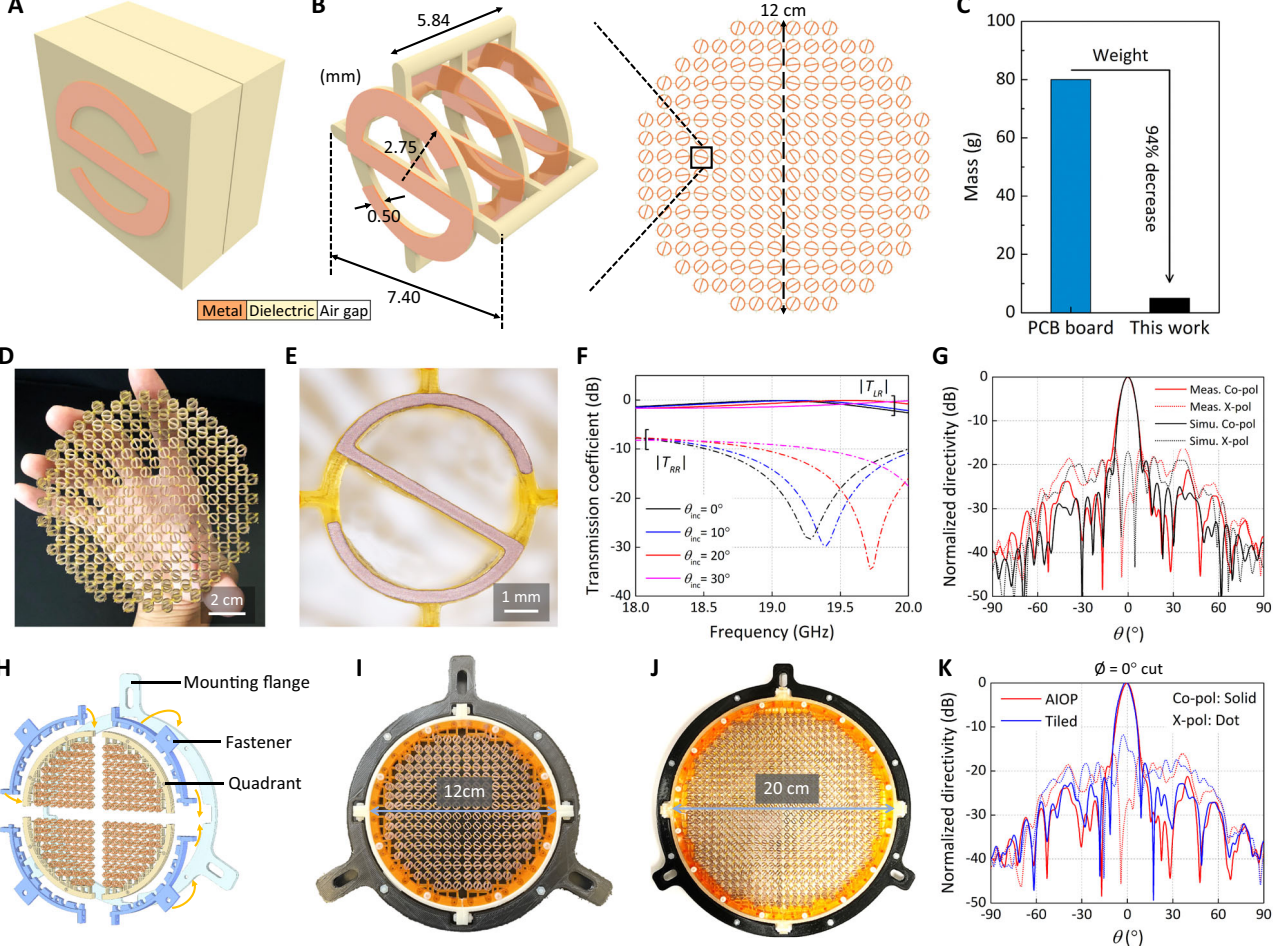
Ultralight transmitarray and scalability. **A**, **B** Schematic comparison of **A** conventional lithographic transmitarray unit cell with **B** ultralight transmitarray unit cell printed with CPD. **C** Weight comparison between the ultralight transmitarray and a traditional PCB process manufactured transmitarray of a similar design at the same frequency (estimated based on the design in ref. ^36^). **D**, **E** Photos showing the complex metal-dielectric structure of copper and acrylate polymer. **F** The transmission coefficient (|*T*_LR_|: left-hand; |*T*_RR_|: right-hand) of the unit cell under right-hand circularly polarized incidence with different incident angles (*θ*_inc_). **G** Transmitarray simulation (Simu.) and measured (Meas.) results at 19 GHz for the co-polarized (Co-pol) left-hand circularly polarized (LHCP, solid lines) and cross-polarized (X-pol) right-hand circularly polarized (RHCP, dashed lines) components. **H** Horizontally tiling scheme. **I**, **J** The assembly of the 12-cm and 20-cm diameter transmitarray antenna. **K** LHCP (Co-Polarized) and RHCP (Cross-Polarized) experimental data in 0°-cut of AIOP and tiled 12-cm transmitarray at 19 GHz.

Transmitarrays are low-profile and high-gain antennas that are promising for remote sensing and communications through platforms such as CubeSats and SmallSats^2,3^. A typical transmitarray is fed by a low-gain antenna (feed source) and generates highly directive radiation by correcting the spherical phase front of the feed. Phase shifting unit cells, as the building blocks of a transmitarray, are thus the most critical part of a transmitarray design. Most transmitarray unit cells necessitate at least three layers of metallic elements spaced by dielectric to achieve the desired transmission efficiency and phase control. Limited by the traditional subtractive printed circuit board (PCB) manufacturing process, unit cells are designed on copper-plated PCB laminates (e.g., Rogers series) as shown in Fig. 3A. Therefore, the spacing among metallic is inevitably filled by bulky dielectric material, that is not functionally necessary but contributes most of the weight. Besides, the thickness of the dielectric spacing is often constrained to manufacturer standards, which gives away the degree of freedom in the design of the unit cells.

Here we demonstrate the design and printing of a circularly-polarized (CP) 19-GHz ultralight transmitarray antenna. Taking advantage of the CPD process, we innovate the transmitarray unit cell topology and propose an S-ring unit cell (Fig. 3B) that is structurally optimized to minimize the use of dielectric material^36^ (Fig. 3C). The CP transmitarray design is featured with discontinuously distributed conductive S-ring elements throughout the structurally optimized 3D layout where electromagnetic wave phase control is realized through element rotation. This typically allows a wider operational bandwidth than the conventional transmitarray designs that use the elements’ size variation to achieve different phase compensation. The unique 3D structure also promises vast freedom for designing new antennas by freely tuning the air gap and choosing desirable dielectric properties (see Supplementary Materials Fig. S4 and SI 2 and 3 for detailed design principle of the S-ring shape for phase compensation).

To implement the design for direct 3D printing, the structure is divided into metalized and dielectric areas within the digital CAD model, and charged resins are assigned based on their polarity. The printing is achieved by integrating with a fluid handling process to combine the charged and uncharged materials into a single structure^18,37^. The unit cell contains three layers of S-ring elements made of copper and separated by air gap. The dielectric skeleton is included only to support the copper element and to maintain inter-element and inter-layer spacing, Fig. 3D, E. From the transmission coefficient plotted in Fig. 3F, we observe that the 19 GHz transmission coefficient magnitude for the desired left-hand circular polarization (LHCP), |*T*_LR_ | , remained above −0.7 dB at 20° incidence. The transmission phase of the transmitarray unit cell under different incident angles is shown in Fig. S5. Each S-ring element in the transmitarray was properly rotated based on the required phase compensation such that the transmitarray corrects the spherical incident wave front to a planar wave front (for maximum directivity) as shown in Fig. 3B. The dielectric material used here has a stiffness approaching 1 GPa after post-curing showing good structural rigidity, and such material can additionally be composited with carbon fibers^19,38^ or dielectric ceramic powders^33^ to increase mechanical robustness and/or tune dielectric properties. The weight of this printed transmitarray is only 5 g, which is an order of magnitude lower than a 12-cm S-ring transmitarray built with copper-plated laminate (80 g, estimated using Rogers RO3003)^39^, achieving a weight reduction of 94%, as shown in Fig. 3C.

A particular limitation of SLA, and AM in general, is the lateral build-area dimensions, with SLA typically being <10 cm^40^. Increasing the maximum achievable build area and therefore the antenna aperture size would be beneficial for applications requiring high directivity since the directivity scales with the antenna aperture. At 19 GHz, transmitarray with an aperture diameter of around 20 cm can offer desirable antenna directivity for some small satellite applications. Here we demonstrate a modular stackable antenna system via mechanical interlocking mechanisms^41,42^. This modular method offers simple scalability to larger build volumes and allows replaceable sections to mitigate defects while minimizing cost.

We designed and tested snap-fit features to act as interfacial connections and incorporated an external locking frame to tile the antennas (Fig. 3H). This frame assisted in maintaining modular construction integrity and allowed a connection to external aligning mechanisms for system integration. We then printed modular 12-cm and 20-cm transmitarray in 4 quadrants (Fig. 3I, J). The tiled 12-cm transmitarray is the same design as previously shown all-in-one-printing (AIOP) 12-cm transmitarray (Fig. 3B), to directly compare the effects of incorporating tiled assembly (misalignment, defects, etc.) vs. AIOP. Figure 3K shows the measured radiation patterns of the tiled and AIOP transmitarray, revealing minimal effects from the tiling. A 0.2 dB variation in directivity in the tiled (23.9 dBi) vs. the AIOP arrays (24.1 dBi) likely arises from slight misalignment of the pieces. The beam width of the AIOP array in 0°-cut (Fig. 3K) and 90°-cut (Fig. S6) are 7.9° and 8.6°, while the tiled array is 7.9° and 8.2°, respectively. These all indicated that aperture tiling did not noticeably impact the performance of the transmitarray. The 20-cm design exceeds the build area of our printer (19.2 × 12.0 cm^2^) and is only possible to build with assembly of separately printed modules, which highlights the necessity of this tiling mechanism.

### Lightweight horn antenna device

Our CPD method not only allows weight reduction via incorporating lattice structures but also saves weight by taking advantage of the thin film and coating nature of the technique. This holds relevance to horn antennas and waveguide devices that are traditionally built with solid metal using metal machining, injection molding, or AM techniques such as metal laser sintering. A 3D printed horn antenna using binder jetting/sintering or selective laser melting typically has a metallic body that is at least 1 mm thick, giving it considerable weight^43,44^. However, according to the skin effect, the alternating current is distributed dominantly within the thickness of several skin depths (which is several microns at K-band) inside the metal. The rest of the metallic material has minimum effect on the electromagnetic performance of the horn, but rather for mechanical strength. To reduce the weight of such antennas, the remaining material should either be minimized or be as light as possible^45^ (see detailed calculation in Supplementary Information SI 4).

**Fig. 4:**
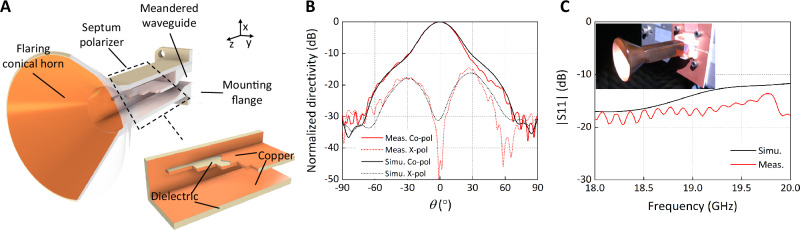
Lightweight horn antenna. **A** CP horn antenna design with complex internal features. Dielectric is shown in tan/gray, and copper in orange. **B** The comparison between the measured and simulated radiation patterns of the horn at 19 GHz. **C** Measured reflection coefficient S11 at the input port of the horn (inset: photo of the printed horn being measured in the spherical near-field range at UCLA).

We present the design and printing of a CP horn antenna at 19 GHz as the feed source for the transmitarray (Figs. 1F and 4A). Distinct from all-metal horns^46–48^ or printed polymer horns entirely coated with conductive materials^45,49^, our horn weighed only 12 g of its dielectric body with a selectively patterned thin layer of copper only on the interior surface where electromagnetic wave propagates. If the same horn were to be built with pure brass (density 8.73 g/mL in volume 7.7 mL)^50^, it would result in more than 5 times heavier weight. The horn consists of complex internal architectures, as shown in Fig. 4A, including a meandered waveguide transition, a square waveguide section with a septum polarizer, a square to circular adapter, and a circular horn section. The horn antenna was designed with a standard WR-42 waveguide interface, making it compatible with a commercially available coax to waveguide adapter (NARDA coax to WR-42 waveguide adapter) for excitation. The septum polarizer is the key section for generating high-purity CP wave. It is constructed by a square waveguide with a thin piece of stepped septum in the center (see dimensions in Fig. S7). One port of the septum polarizer is excited by the meandered waveguide, while the other port is closed with copper (Fig. 1F). The polarizer generates a right-hand circularly polarized (RHCP) wave which is eventually radiated to free space through the circular horn. These complex internal features make the monolithic fabrication of such a horn device unfeasible via traditional techniques but possible via the CPD process demonstrated here.

The printed CP feed horn is measured individually in UCLA’s spherical near-field antenna measurement range (Supplementary Information SI 5 and Fig. S8). The measured radiation patterns at 19 GHz are presented in Fig. 4B. Compared with the simulated results, excellent agreement in the patterns was reached. The measured broadside axial ratio is 0.1 dB, indicating a good circular polarization purity. The simulated directivity of the horn is 15.2 dBi, and the measured directivity is 15.4 dBi. The mismatch loss at the feeding port of the horn was only 0.1 dB at 19 GHz, characterized using the measured reflection coefficient (*S*_11_) as shown in Fig. 4C. These results suggested that the horn was accurately printed and can serve as the feed source for the transmitarrays. The gain of the horn is measured to be 13.8 dB, corresponding to a gain loss of 1.6 dB, which is comparable to similar manufacturing techniques and can be improved by further increasing the copper thickness.

### All 3D printed antenna systems

**Fig. 5:**
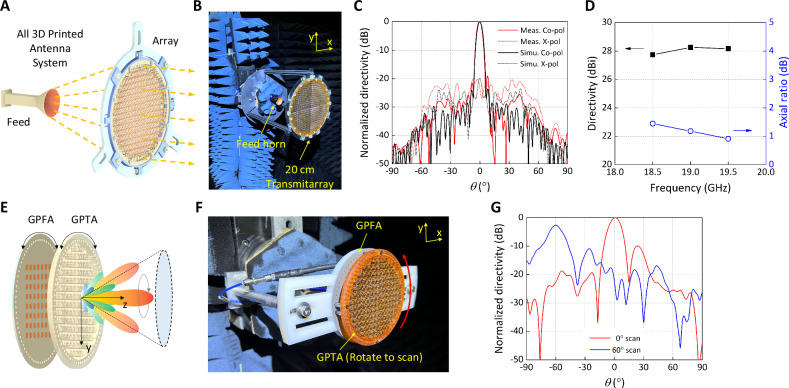
3D printed antenna systems. **A** Schematic for all 3D printed antenna system consisted of a horn antenna and a transmitarray. **B** Photo of the assembled 20-cm transmitarray being measured. **C** The comparison between the simulated pattern and the measured pattern of the 20-cm transmitarray, at 19 GHz (solid line: the co-polarized LHCP pattern; dashed line: the cross-polarized RHCP pattern). **D** The measured directivity and axial ratio of the 20-cm transmitarray over frequency. **E** Schematic for a beam steerable RPA comprised of a gradient-phase transmitarray (GPTA) and a gradient-phase feed array (GPFA). **F** Photo of the printed RPA being measured for its radiation pattern. **G** Representative measured RPA patterns showing beams at 0° and 60°, when using different panel orientations.

We then combined our CPD-manufactured transmitarray and horn antenna to form an all 3D printed antenna system (Fig. 5A). The manufactured transmitarrays by tiling were measured in UCLA’s spherical near-field antenna measurement range (Fig. 5B for the 20-cm tiled transmitarray and Fig. S9A for the 12-cm tiled transmitarray) using the fabricated horn antenna as the feed source. Representative radiation patterns of the transmitarray system at 19 GHz are compared with the simulated patterns in Fig. 5C and S9B, showing good agreement. Note that the transmitarrays were designed to flip the CP handedness from RHCP at the input to LHCP at the output; thus, the sense of co-polarization and cross-polarization are opposite to the definition used for the feed horn. The simulated directivity of this 20-cm transmitarray is 29.1 dBi at 19 GHz, and the measured directivity was 28.3 dBi. The difference between simulations and measurements for the 20-cm transmitarray is a manifestation of the fabrication tolerance in the horn and the transmitarray. The measured directivity variation of the 20-cm transmitarray is only 0.51 dB within the 18.5 – 19.5 GHz range and the measured axial ratio remains below 2 dB throughout the band (Fig. 5D). The ohmic loss in the dielectric and conductor material of both the 12-cm and 20-cm transmitarray are measured to be around 1.0 dB, which is considered reasonable and can be improved by utilizing dielectric resin that specifically target low loss tangent. These indicate that the error introduced by the assembling of tiles is acceptable and mark the successful demonstration of the transmitarrays realized by the four-tile assembly.

The lightweight transmitarray architecture can be implemented to realize other advanced antenna applications such as 2D beam steering, when used as the gradient-phase transmitarray (GPTA) panels of a Risley prism antenna (RPA)^22^. This RPA utilizes the rotation of the GPTA panel and a gradient-phase feed array (GPFA) panel to dynamically control the aperture gradient and consequently the beam scanning angle (Fig. 5E)^22^. A similar 3-layer S-ring unit cell design (Fig. S10) was used to construct the lightweight GPTA, which was then printed (Fig. 1B) and measured in the full RPA system in UCLA’s spherical near-field antenna measurement range (Fig. 5F). The printed GPTA weighs only 28 g (including the mounting frame only for testing purposes), which marks more than 50% weight reduction compared to a same-sized GPTA based on traditional laminate. Some representative radiation patterns of the RPA showing a broadside beam and a 60° scanned beam at 19 GHz are shown in Fig. 5G. These results highlight the various antenna applications that can directly benefit from the CPD technique.

## Discussion

In this work, we have demonstrated a charge-programmed 3D printing method as a versatile and universal platform for fabricating truly ultralight 3D antennas composed of dielectric, conducting phases or their interpenetrating composites. This technology allows for the printing of previously inaccessible electronic architectures and systems via current manufacturing approaches. To meet the requirement of high-frequency antenna, we fine-tuned the printing resins to achieve controllable copper deposition on negative resins with high conductivity and extended the materials of choice to high-performance polymer, wearable elastomer, liquid metal, and ceramic to serve extreme conditions. We designed and printed a class of low-profile, ultralight, and high-gain 19 GHz transmitarray antennas containing arrays of mixed-metal-dielectric unit cells with unique weight-reducing architectures. Enabled exclusively by charge-programmed 3D printing, these structurally optimized unit cells enabled over 90% weight saving compared to traditionally manufactured transmitarray antenna systems while demonstrating full functionalities. A lightweight horn feed with a built-in septum polarizer and meandered waveguide demonstrating a high level of intricacy was also monolithically manufactured using the same process, revealing the versatility of this method in antenna manufacturing. To demonstrate its applicability for industry adoption, we show that these antenna systems can be printed via a desktop, commercially available 3D printing system incorporating the charge-programmed printing scheme. Additionally, we have demonstrated a scaled-up fabrication route via printing modular snap-fit transmitarray antenna components that can be assembled into industry relevant aperture scales. The performance of a fully 3D printed beam-collimating transmitarray antenna system and a beam steerable RPA using 3D printed GPTA have been characterized. The key results such as the measured radiation pattern and directivity are presented. We strongly believe that the outcome of this research work will provide unique opportunities in applying these designs in applications aboard CubeSats and SmallSats whereby the weight is one of the most critical components in selecting the best antenna concepts.

Future improvements of the multi-material printing process, including automated resin exchange and cleaning, can realize additional time, labor, and cost reductions. Nevertheless, the whole fabrication process of each sample can be done within one day, and it is cost-effective. The charge-programmed printing method is compatible with highly elastic to ultra-stiff substrate materials and a variety of multi-functional coating materials beyond metals, including magnetic, nanomaterials, piezoelectric, and more, enabling soft wearable devices and robotic systems. This robust material and manufacturing approach reported could lead to a new approach for custom printing electronic systems at a fraction of weight, a critically important advancement for 5 G/6 G, wearables, satellite communications, aerospace, transportation industry, and beyond.

## Methods

### Materials

Bis(2-(methacrylooyloxy)ethyl)) phosphate (PDD), trimethylpropane triacrylate (TMPTA), polyethylene glycol diacrylate Mn~250 (PEGDA), phenylbis(2,4,6-trimethylbenzoyl)phosphine oxide (Irg819), Sudan (I), tetraaminepalladium (II) chloride monohydrate (Pd+), and borane dimethylamine complex (DMAB), poly(pyromellitic dianhydride-co-4,4′-oxydianiline), amic acid solution (PMDAODA PAA, 15.0-16.0 wt.% in *N*-methylpyrrolidone), 2-(dimethylamino)ethyl methacrylate (DMAEMA), hexahydro-4-methylphthalic anhydride, ethoxylated trimethylolpropane triacrylate, glycidyl methacrylate, and 2,4,6-tris-dimethylaminomethyl phenol (Epikure) were all purchased from Millipore-Sigma. EPON 828 was purchased from Miller-Stephenson. The commercial ultra-low dielectric loss resin was a product of Arkema (N3D-DIELEC731) with a dielectric constant *k* of 2.59 at 10 kHz and loss tangent tan(*δ*) of 0.003 at 10 GHz. Ethylene glycol phenyl ether acrylate (PA) was generously provided by Allnex.

Electroless metal plating solutions were purchased from Caswell Inc. and used as received: Electroless Copper Kit.

### Resin formulations

#### Acrylates

##### Negative resin

PDD and TMPTA in 10/90 – 50/50 wt./wt. ratios, 2 wt.% Irg819 and 0.05 wt.% Sudan I (both in respect to the total mass of the PDD and TMPTA mixture).

##### Neutral (PEGDA) resin

10 g PEGDA, 0.2 g Irg819, 0.005 g Sudan I.

#### Ultra-low dielectric loss material

##### Negative resin

N3D-DIELEC731 and PDD in 90/10 wt./wt. ratio.

##### Neutral resin

N3D-DIELEC731 as received.

#### Polyimide

##### Negative resin

78 g PMDAODA PAA solution, 4.84 g DMAEMA (0.5 equiv. in respect to the COOH groups of the PAA), 4.17 g PDD, and 0.33 g Irg819.

##### Neutral resin

78 g PMDAODA PAA solution, 4.84 g DMAEMA, and 0.33 g Irg819^29^.

After printing, the structure was carefully dried and imidized at 400 °C for 30 min in nitrogen^29^ and then selectively deposited with copper.

#### Epoxy resin

##### Thermal (T) resin

hexahydro-4-methylphthalic anhydride and EPON 828 in a mass ratio of 89/100.

##### Photo (P) resin

ethoxylated trimethylolpropane triacrylate and glycidyl methacrylate in a mass ratio of 95/5 with 2 wt.% Irg819^30^.

##### Negative resin

T resin, P resin, and PDD in a mass ratio of 50:30:20 with 0.1 wt.% Epikure. The resin should be used as soon as possible.

##### Neutral resin

T resin and P resin in a mass ratio of 50:50 with 0.1 wt.% Epikure.

After printing, the structure was thermally cured at 100 °C and 160 °C for 2 h each and then selectively deposited with copper.

#### Elastomer

##### Negative resin

PA and PDD in a mass ratio of 95:5 with 2 wt.% Irg819 and 0.025 wt.% Sudan I.

##### Neutral resin

PA with 2 wt.% Irg819 and 0.025 wt.% Sudan I.

After printing, the structure was selectively deposited with copper. Then the sample was dipped into 1 M HCl solution with a droplet of EGaIn in the bottom. EGaIn spontaneously wet the surface of copper because of the formation of CuGa_2_.

#### Ceramic

##### Negative resin

Lead zirconate titanate (PZT 855, from APC Piezo, USA), PEGDA, and PDD in a volume ratio of 30/60/10 with 2.5 wt.% Irg 819 in respect to the total mass of PEGDA and PDD.

##### Neutral resin

PZT 855 (APC Piezo, USA) and PEGDA in a volume ratio of 35/65 with 2.5 wt.% Irg 819 to PEGDA.

The resins were prepared using a high-energy ball mill EMAX (Retsch) at 1000 rpm for 30 min. After printing, the structure was selectively deposited with copper, sintered at 1000 °C in air^34^, and reduced with nitrogen/hydrogen 95/5 mixture at 300 °C.

### Plating solutions

There should be enough solutions to cover the entire part. Flat bottom and moderately heat resistant plastic containers were used (Ziploc).

#### Pd+ solution

0.2634 g Tetra-amine Palladium Chloride in 250 g deionized water. Solution should be used the same day as made.

#### DMAB solution

0.1475 g DMAB in 250 g deionized water. Solution should be used the same day as made.

#### Electroless copper (Cu)

As per Caswell instruction, 125 g part A and 125 g part B were combined to make 250 g of solution and used within the same day.

### 3D Printing apparatus for CPD

We implement the CPD fabrication of transmitarray, via a desktop stereolithography printer Photon Mono X (ANYCUBIC) integrated with charge programmed resin exchanges to show the ease of fabrication and its adaptability to a desktop system. A single build file was input, and the first material was printed until the layer before the second material should be. At this point, the printer was paused, the resin tray removed, and with the building substrate still attached, the parts were washed with ethanol and dried with Kimwipe. A resin tray with the second material was placed on the printer, and printing resumed. We would note that the precision is largely not affected as the building substrate is not moved. The part was cleaned while fixed on the substrate, and the resin trays were easily interchangeable. A typical antenna part is printed within two hours.

The locking frames of the tiled transmitarray and the horn antenna were printed using the same SLA printer as the transmitarray. The PLA mounting frames were printed by an FDM printer.

### Electroless deposition

Caswell solutions were used as received. The provided “activator” and “sensitizer” solutions were not used. Parts were dipped into DI water and dried on Kimwipe and with pressurized air. They were then dipped into Pd+ solution for 6 min. A transfer pipette was used to remove air bubbles. After 6 min, the part was gently removed and dried with a Kimwipe and pressurized air. No excess liquid should be left on the part. Parts were then placed into DMAB solution and left undisturbed for 5 min. Black deposits should form in the selective plating areas. Parts were then gently removed and dried on Kimwipe and with pressurized air. Parts were then placed in Cu electroless solution and allowed to plate for 10-30 min, but not more than 2 h to prevent cracking. Parts were then dried on Kimwipe, rinsed with DI water, and dried again.

### Antenna simulation

The unit cell’s electromagnetic performance of the transmitarray was evaluated in a full-wave analysis tool (CST Studio), and the geometric parameters of the unit cell were designed for 19-GHz using particle swarm optimization (PSO)^51^. The transmitarray antennas were designed using a MATLAB-based program, and the CAD model was generated in CST studio for full-wave analysis. In simulations, the dielectric material is modeled with a dielectric constant of 3.6 and a loss tangent of 0.02, which are estimated values for the cured resin. However, parametric studies showed the performance of the unit cell is insensitive to the variation of the dielectric constant, meaning that the unit cell can tolerate a reasonable variation of the electric property of the resin.

## Supplementary information

Supplementary Information

Transparent Peer Review file

## Data Availability

All data generated in this study have been deposited in the Figshare database under accession code [10.6084/m9.figshare.26964955].

## References

[1] Guo, Y. J.; Ziolkowski, R. W. *Advanced Antenna Array Engineering for 6G and Beyond Wireless Communications* (Wiley, 2021).

[2] Wang, J., Manohar, V. & Rahmat-Samii, Y. Enabling the Internet of Things With CubeSats: a review of representative beamsteerable antenna concepts. *IEEE Antennas Propag. Mag.* **63**, 14–28 (2021).

[3] Davarian, F. et al. Improving small satellite communications and tracking in deep space—a review of the existing systems and technologies with recommendations for improvement. Part II: small satellite navigation, proximity links, and communications link science. *IEEE Aerosp. Electron. Syst. Magn.* **35**, 26–40 (2020).

[4] Papathanasopoulos, A., Budhu, J., Rahmat-Samii, Y., Hodges, R. E. & Ruffatto, D. F. 3-D-printed shaped and material-optimized lenses for next-generation spaceborne wind scatterometer weather radars. *IEEE Trans. Antennas Propag.* **70**, 3163–3172 (2022).

[5] Budhu, J. et al. Three-dimensionally printed, shaped, engineered material inhomogeneous lens antennas for next-generation spaceborne weather radar systems. *IEEE Antennas Wirel. Propag. Lett.* **17**, 2080–2084 (2018).

[6] Zhu, D. Z., Gregory, M. D., Werner, P. L. & Werner, D. H. Fabrication and characterization of multiband polarization independent 3-D-printed frequency selective structures with ultrawide fields of view. *IEEE Trans. Antennas Propag.* **66**, 6096–6105 (2018).

[7] Cheng, Q. et al. Dual circularly polarized 3-D printed broadband dielectric reflectarray with a linearly polarized feed. *IEEE Trans. Antennas Propag.* **70**, 5393–5403 (2022).

[8] Veljovic, M. J. & Skrivervik, A. K. Circularly polarized axially corrugated feed horn for cubesat reflectarray applications. In *2020 14th European Conference on Antennas and Propagation (EuCAP)*, 15-20 March 2020; pp 1-4. (2020).

[9] García-Marín, E., Masa-Campos, J. L., Sánchez-Olivares, P. & Ruiz-Cruz, J. A. Evaluation of additive manufacturing techniques applied to ku-band multilayer corporate waveguide antennas. *IEEE Antennas Wirel. Propag. Lett.* **17**, 2114–2118 (2018).

[10] MacDonald, E. & Wicker, R. Multiprocess 3D printing for increasing component functionality. *Science* **353**, aaf2093 (2016). 27708075 10.1126/science.aaf2093

[11] Elsallal, M. W., Hood, J. R. & Kindt, R. Development of substrate-free frequency-scaled ultra-wide spectrum element (FUSE) phased array. In *2016 IEEE International Symposium on Phased Array Systems and Technology (PAST)*, 18-21 Oct. 2016, pp 1-5 (2016).

[12] Zhu, J. et al. Additively manufactured millimeter-wave dual-band single-polarization shared aperture fresnel zone plate metalens antenna. *IEEE Trans. Antennas Propag.* **69**, 6261–6272 (2021).

[13] Zhu, J., Yang, Y., Wang, F., Lai, J. & Li, M. 3D printed spin-decoupled transmissive metasurfaces based on versatile broadband cross-polarization rotation meta-atom. *Adv. Opt. Mater.* **11**, 2202416 (2023).

[14] Li, M. & Yang, Y. Single- and multiple-material additively manufactured electronics: a further step from the microwave-to-terahertz regimes. *IEEE Microw. Mag.* **24**, 30–45 (2023).

[15] Adams, J. J. et al. Conformal printing of electrically small antennas on three-dimensional surfaces. *Adv. Mater.* **23**, 1335–1340 (2011). 21400592 10.1002/adma.201003734

[16] Smith, P. J., Shin, D. Y., Stringer, J. E., Derby, B. & Reis, N. Direct ink-jet printing and low temperature conversion of conductive silver patterns. *J. Mater. Sci.* **41**, 4153–4158 (2006).

[17] Zheng, X. et al. Ultralight, ultrastiff mechanical metamaterials. *Science* **344**, 1373–1377 (2014). 24948733 10.1126/science.1252291

[18] Xu, Z. et al. Vat photopolymerization of fly-like, complex micro-architectures with dissolvable supports. *Addit. Manuf.* **47**, 102321 (2021).

[19] Xu, Z. et al. Additive manufacturing of two-phase lightweight, stiff and high damping carbon fiber reinforced polymer microlattices. *Addit. Manuf.* **32**, 101106 (2020).

[20] Gerard, N. J. R. K. et al. Three-dimensional trampolinelike behavior in an ultralight elastic metamaterial. *Phys. Rev. Appl.* **16**, 024015 (2021).

[21] Hensleigh, R. et al. Charge-programmed three-dimensional printing for multi-material electronic devices. *Nat. Electron.* **3**, 216–224 (2020).

[22] Wang, J. & Rahmat-Samii, Y. A simplified configuration of beam-steerable risley prism antennas: principles and validation. *IEEE Antennas Wirel. Propag. Lett.* **21**, 2288–2292 (2022).

[23] Zheng, X. et al. Multiscale metallic metamaterials. *Nat. Mater.* **15**, 1100–1106 (2016). 27429209 10.1038/nmat4694

[24] Yang, C. L., Tsai, C. L. & Chen, S. H. Implantable high-gain dental antennas for minimally invasive biomedical devices. *IEEE Trans. Antennas Propag.* **61**, 2380–2387 (2013).

[25] Johnson, K. et al. A copper pyramidal fractal antenna fabricated with green-laser powder bed fusion. *Prog. Addit. Manuf.* **7**, 931–942 (2022).

[26] Wang, Z. et al. Charge-programmable photopolymers for 3d electronics via additive manufacturing. *Adv. Funct. Mater*. 10.1002/adfm.202313839 (2024).

[27] Skylar-Scott, M. A., Mueller, J., Visser, C. W. & Lewis, J. A. Voxelated soft matter via multimaterial multinozzle 3D printing. *Nature* **575**, 330–335 (2019). 31723289 10.1038/s41586-019-1736-8

[28] Rana, M. M.; Khanom, R.; Rahman, M. M. Design and Analysis of Vivaldi Antennas. In *2018 International Conference on Innovation in Engineering and Technology (ICIET)*, 27-28 Dec. 2018, pp 1-5 (2018).

[29] Herzberger, J., Meenakshisundaram, V., Williams, C. B. & Long, T. E. 3D printing all-aromatic polyimides using stereolithographic 3D printing of polyamic acid salts. *ACS Macro Lett.* **7**, 493–497 (2018). 35619348 10.1021/acsmacrolett.8b00126

[30] Kuang, X. et al. High-speed 3D printing of high-performance thermosetting polymers via two-stage curing. *Macromol. Rapid Commun.* **39**, 1700809 (2018). 10.1002/marc.201700809 29383797

[31] Yamagishi, K., Zhou, W., Ching, T., Huang, S. Y. & Hashimoto, M. Ultra-deformable and tissue-adhesive liquid metal antennas with high wireless powering efficiency. *Adv. Mater.* **33**, 2008062 (2021). 10.1002/adma.202008062 34031936

[32] Huff, G. H. et al. A physically reconfigurable structurally embedded vascular antenna. *IEEE Trans. Antennas Propag.* **65**, 2282–2288 (2017).

[33] Cui, H. et al. Design and printing of proprioceptive three-dimensional architected robotic metamaterials. *Science* **376**, 1287–1293 (2022). 35709267 10.1126/science.abn0090

[34] Lu, H. et al. 3D Printing and processing of miniaturized transducers with near-pristine piezoelectric ceramics for localized cavitation. *Nat. Commun.* **14**, 2418 (2023). 37105973 10.1038/s41467-023-37335-w PMC10140030

[35] Levine, E. Overview of GPS antennas. In *2009 IEEE International Conference on Microwaves, Communications, Antennas and Electronics Systems*, 9-11 Nov. 2009, pp 1-4. (2009).

[36] Wang, J. et al. Fully 3D-printed lightweight combination of a circularly polarized transmitarray and a feed horn. In *2022 IEEE International Symposium on Antennas and Propagation and USNC-URSI Radio Science Meeting (AP-S/URSI)*, 10-15 July 2022, pp 645-646 (2022).

[37] Chen, D. & Zheng, X. Multi-material additive manufacturing of metamaterials with giant, tailorable negative Poisson’s ratios. *Sci. Rep.* **8**, 9139 (2018). 29904093 10.1038/s41598-018-26980-7 PMC6002359

[38] Hsieh, M.-T. et al. Stiff and strong, lightweight bi-material sandwich plate-lattices with enhanced energy absorption. *J. Mater. Res.* **36**, 3628–3641 (2021).

[39] Papathanasopoulos, A., Wang, J. & Rahmat-Samii, Y. Transmitarray antenna generating circularly polarized orbital angular momentum (OAM) beams: synthesis, prototyping and measurements. In *2021 Antenna Measurement Techniques Association Symposium (AMTA)*, 24-29 Oct. 2021, pp 1-4 (2021).

[40] Wang, C. et al. A general method to synthesize and sinter bulk ceramics in seconds. *Science* **368**, 521–526 (2020). 32355030 10.1126/science.aaz7681

[41] Cheung, K. C. & Gershenfeld, N. Reversibly assembled cellular composite materials. *Science* **341**, 1219–1221 (2013). 23950496 10.1126/science.1240889

[42] Hiller, J. D., Miller, J. & Lipson, H. Microbricks for three-dimensional reconfigurable modular microsystems. *J. Microelectromechanical Syst.* **20**, 1089–1097 (2011).

[43] Zhang, B. et al. Metallic 3-D printed antennas for millimeter- and submillimeter wave applications. *IEEE Trans. Terahertz Sci. Technol.* **6**, 592–600 (2016).

[44] Zhang, B. & Zirath, H. Metallic 3-D printed rectangular waveguides for millimeter-wave applications. *IEEE Trans. Compon., Packaging Manuf. Technol.* **6**, 796–804 (2016).

[45] Timbie, P. T., Grade, J., Weide, D. V. D., Maffei, B. & Pisano, G. Stereolithographed MM-wave corrugated horn antennas. In *2011 International Conference on Infrared, Millimeter, and Terahertz Waves*, 2-7 Oct. 2011, pp 1-3 (2011).

[46] Chen, M. H. & Tsandoulas, G. A wide-band square-waveguide array polarizer. *IEEE Trans. Antennas Propag.* **21**, 389–391 (1973).

[47] Huang, Y., Geng, J., Liang, X., Jin, R. & Bai, X. A novel CP horn antenna with switchable polarization by single port feeding. *Int. J. Antennas Propag.* **2015**, 562521 (2015).

[48] Jazani, G. Abbas P. Design of dual‐polarised (RHCP/LHCP) quad‐ridged horn antenna with wideband septum polariser waveguide feed. *IET Microw, Antennas Propag.* **12**, 1541–1545 (2018).

[49] Wahyudi, A. H., Sumantyo J. T. S., Wijaya, S. & Munir, A. PLA-based 3D printed circularly polarized X-band horn array antenna for CP-SAR sensor. In *2018 International Workshop on Antenna Technology (iWAT)*. 05-07 March 2018; 10.1109/IWAT.2018.8379219 (2018).

[50] Hossain, S. D.; Lohani, B.; Price, R. M.; Roberts, R. C. Metal Additive Fabrication and Characterization of Horn Antenna. In *2022 IEEE Texas Symposium on Wireless and Microwave Circuits and Systems (WMCS)*, 19-20 April 2022, pp 1-4 (2022).

[51] Robinson, J. & Rahmat-Samii, Y. Particle swarm optimization in electromagnetics. *IEEE Trans. Antennas Propag.* **52**, 397–407 (2004).

