# Supplementary material for: Ultra-light antennas via charge programmed deposition additive manufacturing
Source: Nat Commun. 2025 Jan 8;16:427. doi: 10.1038/s41467-024-53513-w (PMC11711757; doi:10.1038/s41467-024-53513-w)
Supplement: Supplementary file 1 — Supplementary Information [file 41467_2024_53513_MOESM1_ESM.pdf]

Supporting Information

Ultra-Light Antennas via Charge Programmed Deposition Additive Manufacturing

Authors: Zhen Wang,[1,2†] Ryan Hensleigh,[2†] Zhenpeng Xu,[1,2†] Junbo Wang,[3†] James JuYoung Park,[1] Anastasios Papathanasopoulos,[3] Yahya Rahmat-Samii,[3*] Xiaoyu (Rayne) Zheng[1,2,4*]

Author Affiliations:

[1]Advanced Manufacturing and Metamaterials Laboratory, Department of Material Science and Engineering, University of California, Berkeley, California 94720, USA

[2]Department of Civil and Environmental Engineering, University of California, Los Angeles, California 90095, USA

[3]Department of Electrical and Computer Engineering, University of California, Los Angeles, California 90095, USA

[4]Lawrence Berkeley National Laboratory, Berkeley, California 94720, USA

†: Authors contributed equally to this work

***Corresponding author:**
X. Zheng, Email: rayne23@berkeley.edu; Y. Rahmat-Samii, Email: rahmat@ee.ucla.edu

Contents

SI. 1. Multi-material 3D printing 1
Figure S1. 2
Figure S2. 3
Figure S3. 4
SI. 2. Design of the 'S'-shaped ring 4
Figure S4. 6
Figure S5. 6
SI. 3. Phase compensation 7
Figure S6. 8
SI. 4. Skin effect weight reduction 9
Figure S7. 10
SI. 5. Radiation pattern measurement 11
Figure S8. 12
Figure S9. 13
Figure S10. 13
Table S1 14
References 15

SI. 1. Multi-material 3D printing

The transimitarray presented in this work was printed via a commercial stereolithography printer via manual resin exchange. The fabrication precision is not affected as the building substrate is not moving, and the printed part is cleaned while fixed on the substrate during material switching. While this exchange process is quite long, usually taking ~5 min.

In our previous works[1,2], we developed microfluidic methods to automatically clean and exchange reins during printing. Although the time per material exchange is reduced to about 30 seconds, these methods as well as other reported techniques[3,4] are limited to a small printing size (length less than 4 cm). While the transimitarray usually has a size larger than 10 cm.

To resolve the aforementioned difficulties, we are developing a large-scale multi-material 3D printing system integrated with a fluidic system to automatically clean and exchange materials. As shown in Fig. S1a, custom multi-material channels were designed to enable the switch of resins and ethanol (which cleans the previous material). Peristaltic pumps, pipes, and nozzles were used to enable the extrusion of the multiple resins. If another material needs to be printed in the next layer, the locking gate lifts, allowing the resin to flow into the recycling vat. To avoid contamination between resins, ethanol was used to clean uncured resins off solidified parts and the printing vat, followed by air blowing to dry the ethanol residual. The process was repeated layer by layer, combining multiple materials into a 3D structure. Fig. S1b shows our current setup, where the black vat in the middle is designed to facilitate resin switching. Fig. S1c demonstrates an as-fabricated large multi-functional material (PEGDA, charged resin) sample as a transmitarray (3D antenna) after being selectively deposited with a copper layer. We are planning a future publication further detailing this method.

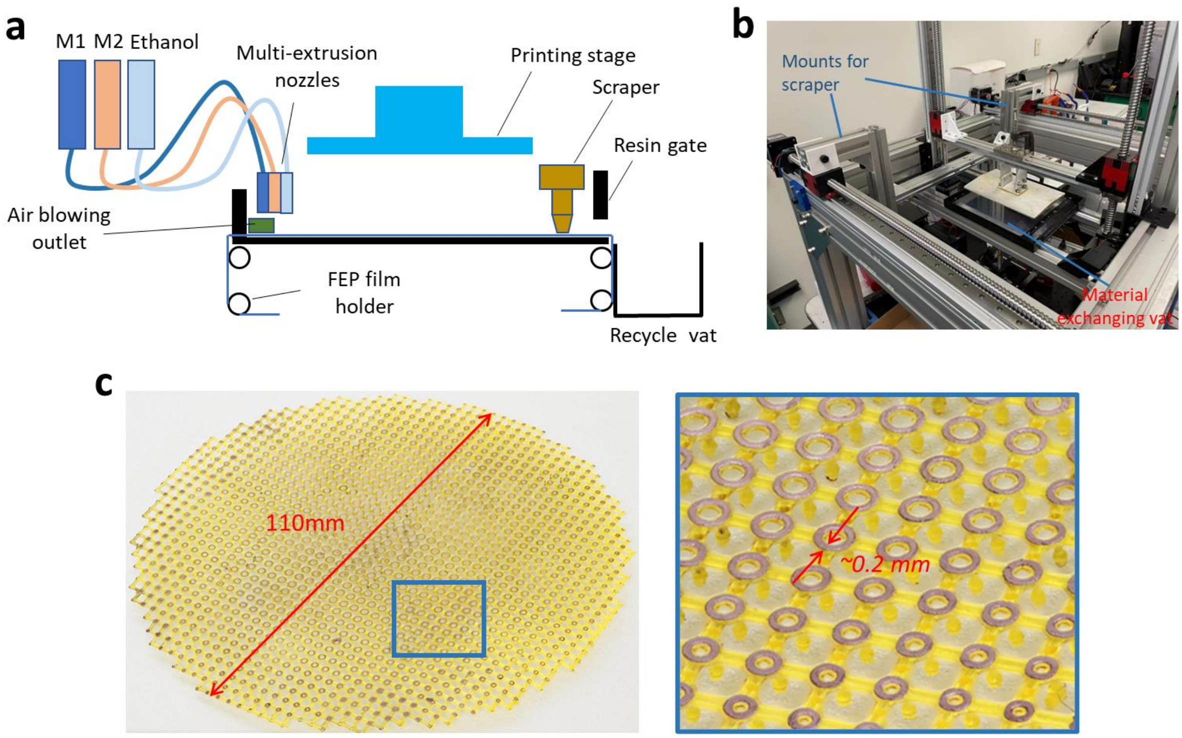

Figure S1. (a) Schematic of the multi-material exchanging system, cleaning system, and resin recycling system. (b) The overall system setup. (c) An antenna array made of two different resins and copper was deposited onto the charged resin.

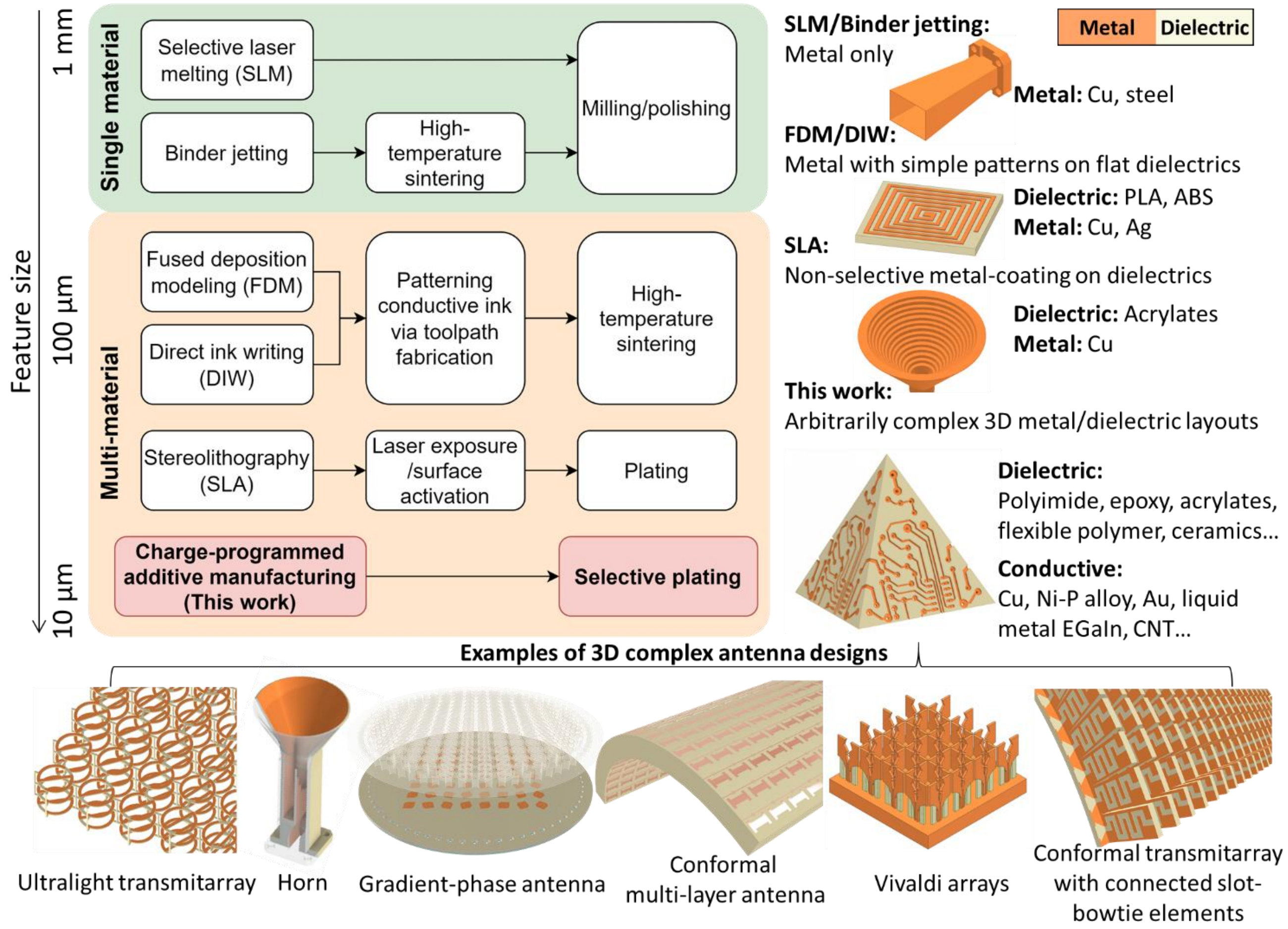

Figure S2. Comparison between this work and conventional 3D printing techniques in fabricating complex multi-material antennas, such as ultralight transmitarray antenna with metallic/dielectric lattice and arrays (focus radio waves into high-gain beams by phase shifting), advanced horn device with complex internal conductive and dielectric paths (create high-purity circular polarization in wide bandwidths), gradient phase antenna (beam steering), conformal antenna (wide angle coverage), and Vivaldi antenna arrays (featured with broadband characteristics).

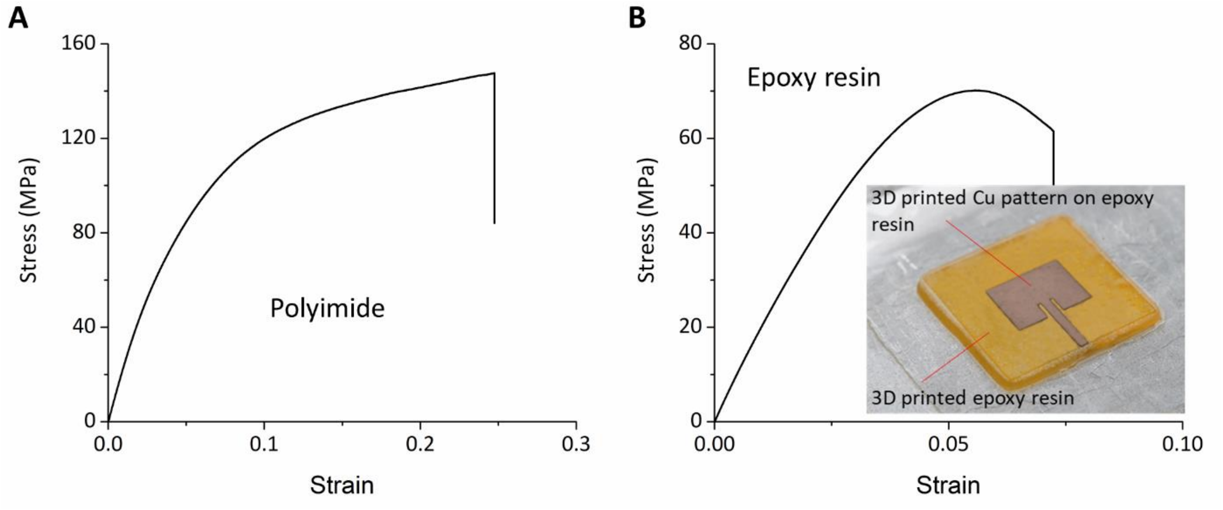

Figure S3. Tensile stress-strain curves of 3D printed (A) polyimide and (B) epoxy resin.

SI. 2. Design of the 'S'-shaped ring

The role of the transmitarray unit cell is to provide high transmission to the desired polarization component and to provide controllable phase shift (desirably covering the range of 0-360°) to the transmitted wave. Transmitarray unit cell was traditionally realized using stacked layers of square conductor patches spaced by dielectric laminate. Such designs achieve variable phase shift by varying the resonant frequency of the element, which is done by varying the dimension of the patches. This method makes the unit cell inherently narrow band due to the limited overlap bandwidth among different elements. Our S-ring unit cell, in contrast, realizes phase control through element rotation. The phase shift of the transmitted wave is twice the angle of rotation of the elements (according to the geometrical phase property[5]), and thus no variation in the element dimension is required. This property makes this type of unit cells naturally suitable for a wider operational bandwidth. Although bandwidth was not the major concern for this S-ring unit cell, it does achieve a very competitive 1-dB transmission bandwidth (1.2 GHz, or 6.3% fractional

bandwidth at 19 GHz) compared to traditional transmitarray unit cells. The constituent transmitarrays also achieved promising directivity bandwidth.

Because of the skin effect, the electromagnetic current is distributed within the thickness of only several skin depths underneath the surface (at 19 GHz the skin depth is 0.47 μm for copper). The ohmic loss due to the conductor is thus very minor. The ohmic loss in traditional transmitarrays are dominantly contributed by the bulky dielectric substrate, which is largely avoided in our transmitarray design thanks to the skeleton-supported unit cell configuration. In other words, the unit cell configuration in this work not only reduces weight, but also avoids excessive ohmic loss in the dielectric material.

The S-ring unit cell concept is carried from the previous development at UCLA[6]. The geometry of the S-ring is determined by the parameters shown below in Fig. S4. The element spacing P is usually fixed to be (or smaller than) half-wavelength in the operational band to avoid any grating lobes. In this case we chose P = 7.4 mm. The outer diameter of the ring (R_{out}), the opening angle of the ring (α), and the spacing between adjacent layers (t) are most critical to the performance of the unit cell. These three parameters were optimized using the built-in particle swarm optimizer in CST. The design goal was set to maximize the magnitude of the transmission coefficient ($|T_{LR}|$) at 19 GHz since bandwidth was not the major consideration for this demonstration. The trace width w does not play a significant role in the unit cell performance, and it was made to be 0.5 mm as a reasonable width for ease of fabrication.

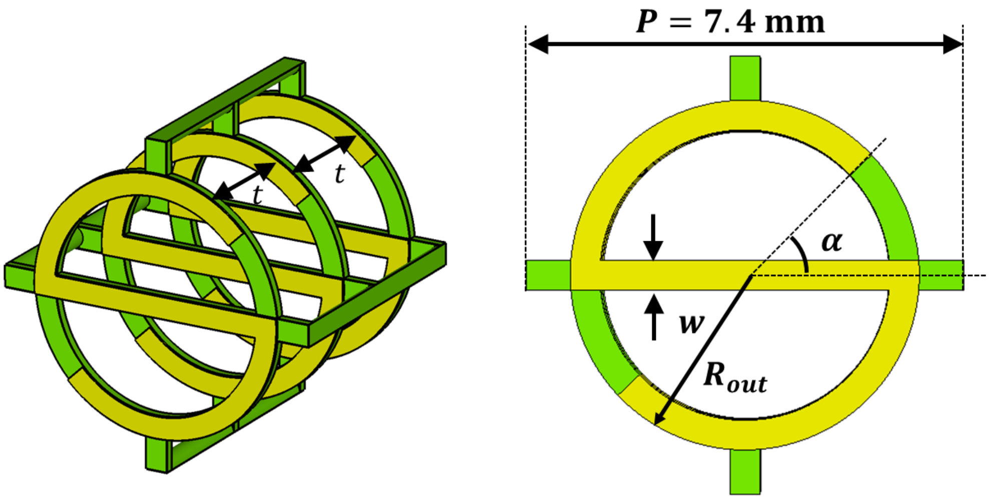

Figure S4. The parameters for designing the S-ring unit cell element.

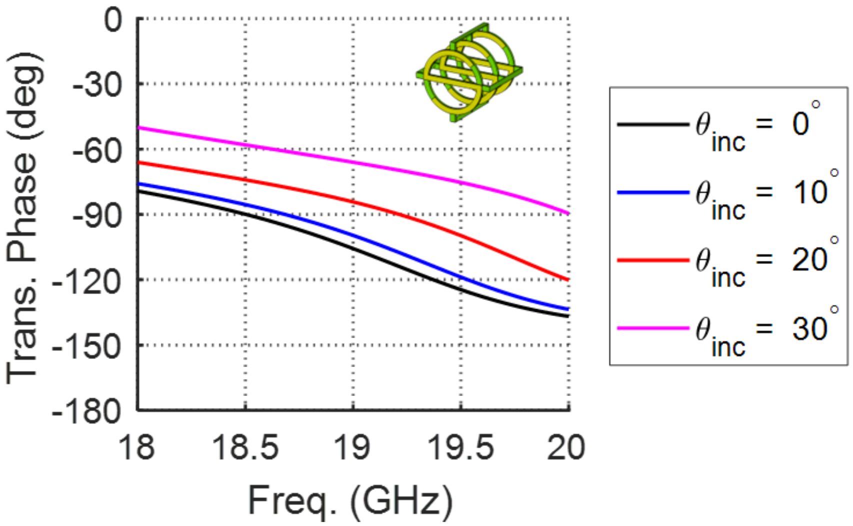

Figure S5. The simulated unit cell transmission phase versus incident angle.

SI. 3. Phase compensation

The role of the transmitarray is to provide high transmission to the wave of certain polarization and to provide the proper transmission phase compensation to turn a spherical incident wavefront to a planar output wavefront to maximize directivity. The center of the spherical wavefront is the focal point of the transmitarray, which is also where the phase center of feed source should be placed at. With the focal length F and the locations of each unit cell (x_i, y_i, z_i) determined, the incident phase for i^{th} unit cell can be calculated as:

$$\Psi_{inc}(x_i, y_i, z_i) = -k\sqrt{x_i^2 + y_i^2 + (z_i - F)^2} \quad (1)$$

with k being the free space wavenumber at the center frequency.

The desired output phase is constant across the aperture and can be set to 0 for simplicity. The required phase compensation by the ith unit cell becomes:

$$\Psi_{comp}(x_i, y_i, z_i) = 0 - \Psi_{inc}(x_i, y_i, z_i) \quad (2)$$

With $\Psi_{comp}(x_i, y_i, z_i)$, we then design the required rotation angles for each element. The S-ring unit cell achieves $0° - 360°$ phase compensation through merely the rotation of the S-ring element. This is governed by the geometrical phase property, which has been well-discussed in the literature[7]. Essentially, when the unit cell is excited by RHCP wave, the transmission phase of the LHCP component is twice the angle of rotation of the element. Therefore, the rotation angle for the ith element is then $\Psi_{comp}(x_i, y_i, z_i)/2$. A "map" containing all the unit cell locations and their corresponding rotation angles are then generated. This process was accomplished in MATLAB using customized codes developed at UCLA.

Lastly, the CAD model of the transmitarray was created by assigning each unit cell the proper rotation angles based on the generated "map" in the previous step. This is accomplished using the Macro scripting function in CST Studio with customized codes developed at UCLA. The transmitarray is then ready for full wave simulation.

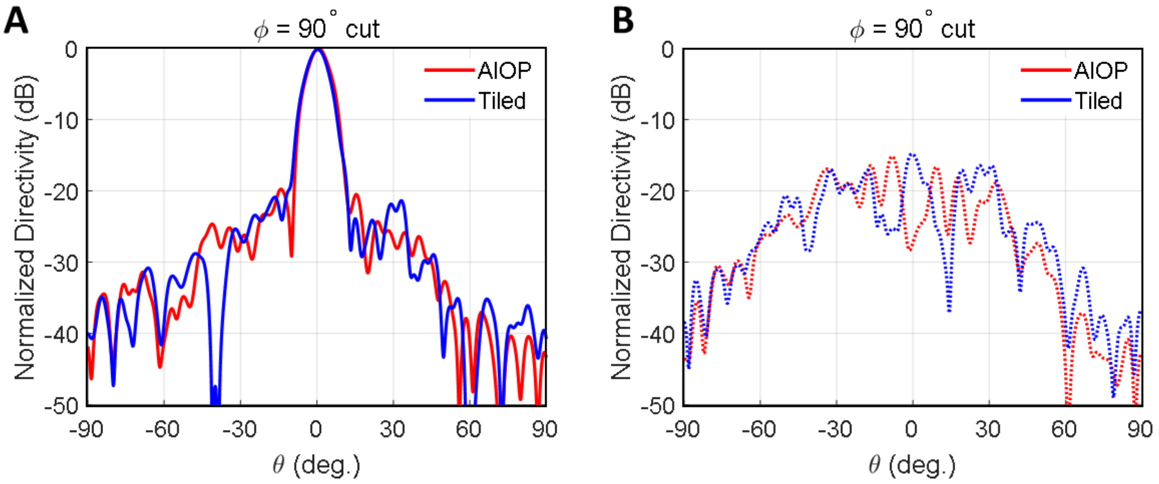

Figure S6. (A) LHCP (Co-Polarized) and (B) RHCP (Cross-Polarized) experimental data in 90°-cut of AIOP and tiled 12-cm transmitarray at 19 GHz

SI. 4. Skin effect weight reduction

Consider two antennas which differ only in their materials. Their volumes are the same, but one, V_m, is fully metal (brass, ρ_m= 8.73 g/mL), while the other, V_s, polymer (ρ_p= 1.1 g/mL) (0.99 mm) with a thin (10 µm) coating of copper (ρ_c= 8.96 g/mL).

$$V_m = V_s \quad (3)$$

$$\rho_m / m_m = \rho_s / m_s \quad (4)$$

$$(8.73 \text{ g/mL})/m_m = (\rho_c + \rho_p)/m_s \quad (5)$$

$$(8.73 \text{ g/mL})/m_m = ((8.96 \frac{g}{mL} * 0.01\, mm) + (1.1 \frac{g}{mL} * 0.99\, mm)/m_s \quad (6)$$

$$\frac{m_s}{m_m} = ((8.96 * 0.01) + (1.1 * 0.99)/(8.73 \text{ g/mL}) \quad (7)$$

$$\frac{m_s}{m_m} = 0.135 \quad (8)$$

The mass of our copper coated on polymer, skin antenna (m_s) with density (ρ_s) will inherently be much lighter than the pure brass antenna (m_m), only 13.5% of the mass.

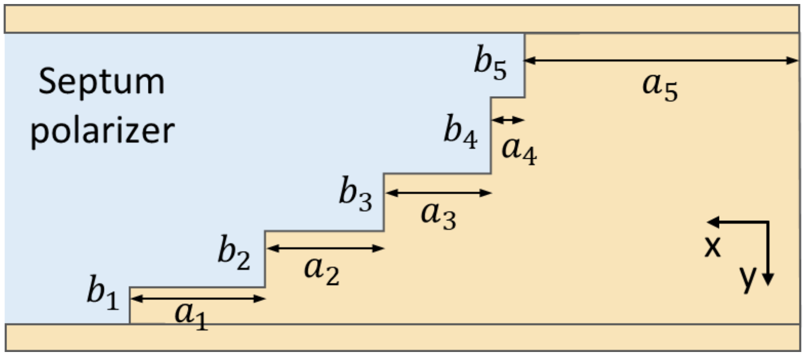

Figure S7. Schematic of the designed septum polarizer (a_1-a_5: 5.7, 5.0, 4.5, 1.4, 11.6 mm; b_1-b_5: 11.4, 2.0, 2.1, 2.9, 2.3 mm).

SI. 5. Radiation pattern measurement

The radiation pattern is measured using the NSI-2000 spherical near-field antenna measurement range at UCLA. A schematic of the key components of the measurement setup is provided as in Figure S6. During measurement, the vector network analyzer (VNA) transmits signal from its port 1 to the antenna under test (AUT), which in our case is the "horn + transmitarray" system or the horn alone. In either case, the horn is excited as the only radiating source that has a spherical wavefront.

The AUT's radiation is captured by an open-ended waveguide (OEWG) probe that acts as the receiver. The received signal is then sent to port 2 of the VNA. The AUT is rotated about two axes during measurements, which allows the probe to capture the electric field on a sphere that encloses the AUT. The entire measurement process and the post-processing procedures (e.g., near-field to far-field transformation) are performed automatically by the NSI-2000 software package. Far-field radiation patterns and directivity numbers are reported by the software.

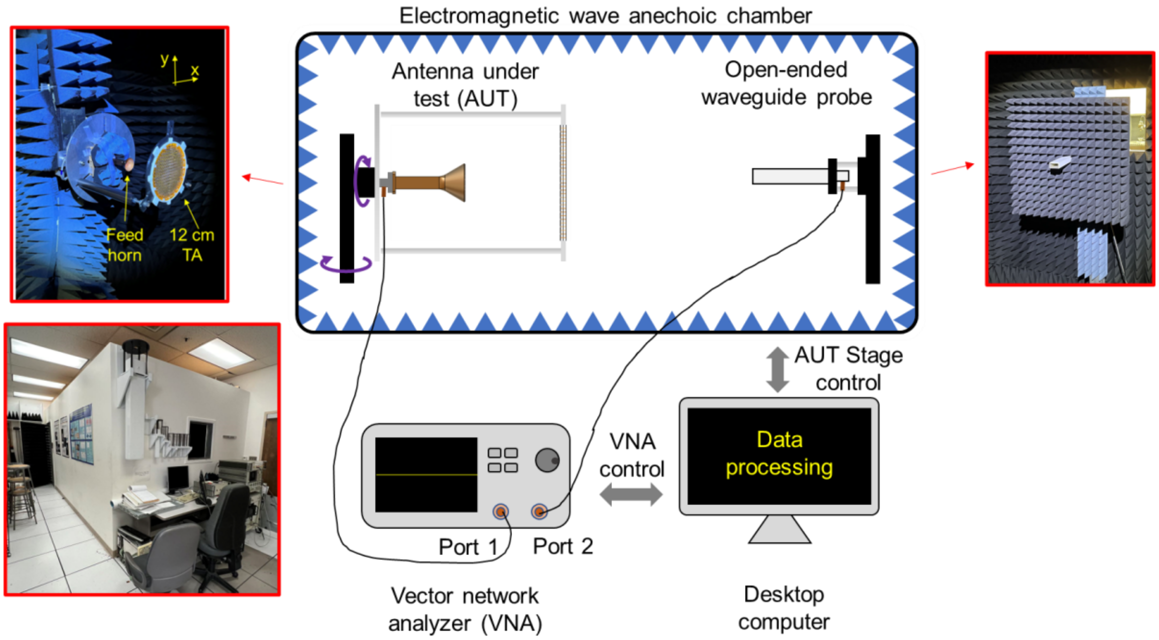

Figure S8. The schematic of the key components of the antenna measurement setup and some photos taken outside and inside the NSI-2000 anechoic range at UCLA.

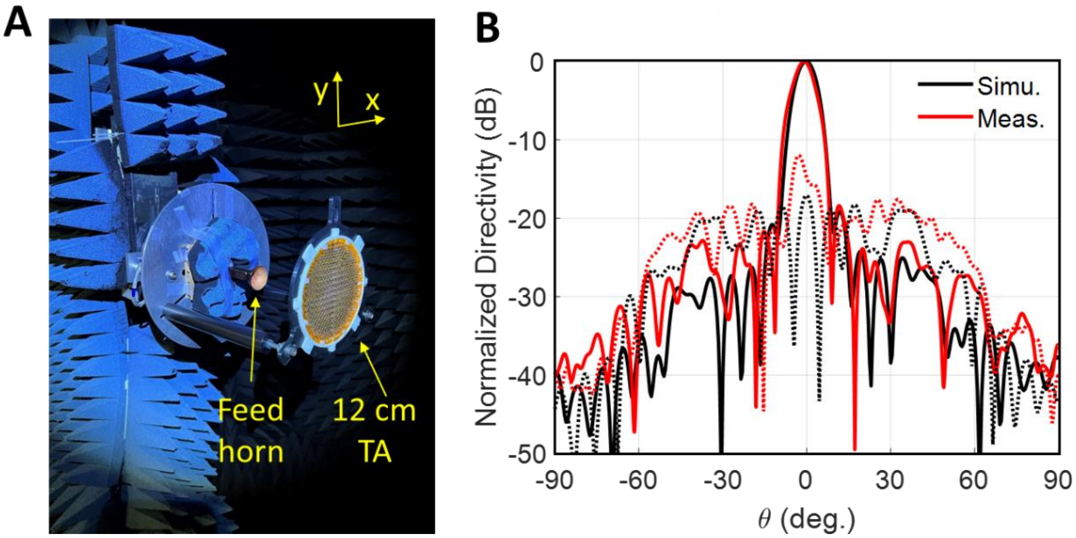

Figure S9. (A) Photo of the tiled 12-cm transmitarray being measured. (B) The measured and simulated patterns of the tiled 12-cm transmitarray at 19 GHz. The simulated directivity of this 12-cm transmitarray at 19 GHz is 24.7 dBi, and the measured directivity of the tiled 12-cm transmitarray is 23.9 dBi.

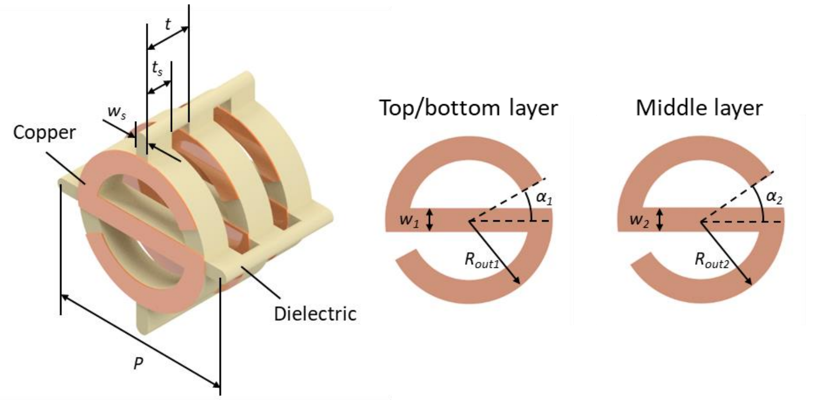

P	R_{out1}	w_1	α_1	R_{out2}	w_2	α_2	t	t_s	w_s
6.60 mm	2.65 mm	0.77 mm	28.2°	2.78 mm	0.82 mm	32.3°	1.75 mm	1.0 mm	0.50 mm

Figure S10. The dimensions of the three layers of S-ring array of the GPTA.

Table S1. Antenna manufacturing technologies comparison table

Method	Method description	Manufacturing time & sample volume	Demonstrated Sample Volume	Minimal achieved feature size	Achievable structure complexity	Material types	Post treatment	Paper
Charge programmed method (this work)	Multi-material stereolithography and selective deposition	2 hours for a 3-layer transmitarray design (1.5 hours printing time, 0.5 hour plating time)	$20 \times 20 \times 1$ cm^3	0.05 mm	Arbitrary 3D lattice-like structures	Polymer, composite, metal, ceramic, nanomaterial	None (room temperature water based chemical deposition)	This work
Binder jetting	Binder jetting and sintering	4 hours for printing and 24 hours for sintering (estimated)	$2 \times 2 \times 5$ cm^3	1.82 mm	Arbitrary 3D lattice-like structures	Stainless steel	Sinter of metals	B. Zhang, IEEE Transactions on Terahertz Science and Technology 2016, 6, 592.
Stereolithography	Molding with regular stereolithography	30 hours	$2 \times 2 \times 2$ cm^3	0.28 mm	Arbitrary 3D lattice-like structures, but only for dielectric	Polymer, conductive ink	Sinter of conductive ink	E. Macdonald, IEEE Access 2014, 2, 234.
FDM with traditional techniques	FDM and assembly with antenna wires	10 hours (estimated)	$8 \times 8 \times 4$ cm^3	1.27 mm	3D structures for dielectric and 2D structures for metal	Polymer, metal	Assembly of metal	J. M. Oy of metal dielectric and 2D structures for metal tricgy 2016, 6, 592
FDM	FDM and micro-dispensing of conductive materials	24 hours or more (estimated)	$10 \times 10 \times 1$ cm^3	0.2 mm	Arbitrary 2D patterns with pre-defined 3D structures	Polymer, silver paste	None	T. P. Ketterl, IEEE Transactions on Microwave Theory and Techniques 2015, 63, 4382.
Polymer jetting	Material jetting	4 hours	$12 \times 12 \times 12$ cm^3	0.2 mm	Arbitrary 3D lattice-like structures	Single polymer	Removal of support by water and assembly	M. Liang, IEEE Transactions on Antennas and Propagation 2014, 62, 1799.
Direct Digital Manufacturing	Microdispensing, material extrusion and micro-milling	5 hours (estimated)	$5 \times 5 \times 5$ cm^3	0.05 mm	3D structures, but usually limited by toolpath	Polymer, conductive ink (proper rheology is required)	Sinter of conductive ink	https://www.nscrypt.com/printed-antennas/
Membrane projection lithography	Two-photon SLA, e-beam lithography, and 5 steps of directional evaporations of metal	2 h (estimated), 6 microns thick over an unknown area on a silicon wafer	Not reported	700 nm	Array of 3D hollow cubic structure with simple 2D patterns on each inner surface of the cubic	Polymer, metal	None	D. B. Burckel, J. R. Wendt, G. A. Ten Eyck, J. C. Ginn, A. R. Ellis, I. Brener, M. B. Sinclair, Advanced Materials 2010, 22, 5053.
FDM	FDM and ink jetting of conductive materials with post thermal treatment	3 hours (estimated)	d: 6.5 cm<br>t: 1.15 mm	0.42 mm	3D structures stacked with 2D patterns	Polymer, silver paste	Near infrared sintering	J. Zhu et al., IEEE Transactions on Antennas and Propagation, 2021, 69, 6261. (A)<br>M. Li and Y. Yang, IEEE Microwave Magazine, 2023, 24, 30. (C)
FDM	FDM and molded ink infusion of conductive materials with thermal treatment	5 hours or more (estimated)	$60 \times 60 \times 2.585$ mm^3	0.11 mm	3D bulky structures with penetrating vias	Polymer, silver paste	Near infrared sintering at 140-170 g	Zhu, J. et al., *Adv. Optical Mater.* 2023, 2202416. (B)

References

1. Xu, Z. P. et al. Additive manufacturing of two-phase lightweight, stiff and high damping carbon fiber reinforced polymer microlattices. Addit Manuf 32, 101106 (2020).
2. Chen, D. & Zheng, X. Y. Multi-material Additive Manufacturing of Metamaterials with Giant, Tailorable Negative Poisson's Ratios. Scientific Reports 8, 9139 (2018).
3. Han, D., Yang, C., Fang, N. X. & Lee, H. Rapid multi-material 3D printing with projection micro-stereolithography using dynamic fluidic control. Addit Manuf 27, 606-615, doi:10.1016/j.addma.2019.03.031 (2019).
4. Wang, Q. M. et al. Lightweight Mechanical Metamaterials with Tunable Negative Thermal Expansion. Physical Review Letters 117, 175901 (2016).
5. P. Naseri, S. A. Matos, J. R. Costa, and C. A. Fernandes, “Phase delay versus phase-rotation cells for circular polarization transmitarrays—application to satellite ka-band beam steering,” IEEE Trans. Antennas Propag., vol. 66, no. 3, pp. 1236–1247, 2018.
6. A. Papathanasopoulos, J. Wang and Y. Rahmat-Samii, "Transmitarray Antenna Generating Circularly Polarized Orbital Angular Momentum (OAM) Beams: Synthesis, Prototyping and Measurements," 2021 Antenna Measurement Techniques Association Symposium (AMTA), Daytona Beach, FL, USA, 2021, pp. 1-4, doi: 10.23919/AMTA52830.2021.9620701.
7. P. Naseri, S. A. Matos, J. R. Costa, and C. A. Fernandes, “Phase delay versus phase-rotation cells for circular polarization transmitarrays—application to satellite ka-band beam steering,” IEEE Trans. Antennas Propag., vol. 66, no. 3, pp. 1236–1247, 2018.
