# Supplementary material for: Ultra-light antennas via charge programmed deposition additive manufacturing
Source: Nat Commun. 2025 Jan 8;16:427. doi: 10.1038/s41467-024-53513-w (PMC11711757; doi:10.1038/s41467-024-53513-w)
Supplement: Supplementary file 2 — Transparent Peer Review file [file 41467_2024_53513_MOESM2_ESM.pdf]

nature portfolio

Peer Review File

Ultra-Light Antennas via Charge Programmed Deposition Additive Manufacturing

Corresponding Author: Professor Xiaoyu Zheng

This manuscript has been previously reviewed at another journal. This document only contains reviewer comments, rebuttal and decision letters for versions considered at Nature Communications.

This file contains all reviewer reports in order by version, followed by all author rebuttals in order by version.

Version 0:

Reviewer comments:

Reviewer #1

(Remarks to the Author)
This manuscript is a revised version transferred from Nature Electronics. The authors have clearly clarified the contribution of this work. I have no more comments.

Open Access This Peer Review File is licensed under a Creative Commons Attribution 4.0 International License, which permits use, sharing, adaptation, distribution and reproduction in any medium or format, as long as you give appropriate credit to the original author(s) and the source, provide a link to the Creative Commons license, and indicate if changes were made.
In cases where reviewers are anonymous, credit should be given to 'Anonymous Referee' and the source.
The images or other third party material in this Peer Review File are included in the article's Creative Commons license, unless indicated otherwise in a credit line to the material. If material is not included in the article's Creative Commons license and your intended use is not permitted by statutory regulation or exceeds the permitted use, you will need to obtain

permission directly from the copyright holder.
To view a copy of this license, visit https://creativecommons.org/licenses/by/4.0/
